# The Role of Individual Capabilities in Maximizing the Benefits for Students Using GenAI Tools in Higher Education

**DOI:** 10.3390/bs15030328

**Published:** 2025-03-07

**Authors:** Jia Qi, Ji’an Liu, Yanru Xu

**Affiliations:** University of Chinese Academy of Sciences, Beijing 101408, China; qijia21@mails.ucas.ac.cn (J.Q.); jian.liu@ucas.ac.cn (J.L.)

**Keywords:** GenAI tools, higher education students, usage behavior, critical thinking, self-directed learning ability, artificial intelligence literacy

## Abstract

Although the adoption and benefits of GenAI (Generative Artificial Intelligence) tools among higher education students have been widely explored in existing studies, less is known about how individual capabilities influence the use of these tools. Drawing on the Information System Success Model (ISSM) and the Expectation–Confirmation Model (ECM), this study examines how students’ capabilities, including critical thinking, self-directed learning ability, and AI literacy, impact the quality of information obtained from GenAI tools. Additionally, it explores the relationships among information quality, student satisfaction, and the intention to continue using GenAI tools in higher education. Survey data from 1448 GenAI tools users in Chinese universities reveal that students with stronger capabilities tend to extract higher-quality information, which in turn fosters their satisfaction with GenAI tools and the intention to continue using these tools. The findings highlight the crucial role of individual capabilities in maximizing the potential of GenAI tools, and it emphasizes the need to cultivate students’ critical thinking, self-directed learning ability, and AI literacy to achieve sustainable success in the GenAI era. Theoretically, this study extends the ISSM and ECM by exploring the impact of students’ capabilities and the mediating role of user satisfaction between information quality and the intention to continue using GenAI tools. Practically, this study provides implications for educators and policymakers to enhance students’ capabilities, thus maximizing the potential benefits of GenAI tools in higher education.

## 1. Introduction

GenAI refers to artificial intelligence systems that can extract and understand instructions provided by users and generate responses to questions within several seconds based on large language models (O’Dea, 2024). The integration of GenAI tools in higher education introduces novel opportunities for developing students’ cognitive skills, such as critical thinking and problem-solving skills, through self-directed learning (Hwang & Chang, 2023; Essel et al., 2024; Jia & Tu, 2024; Wu et al., 2024). For example, GenAI tools can first generate various ideas to help students’ idea generation, from which students are then able to connect these ideas using their own knowledge and understanding. This process requires students to actively engage with the information, which in turn strengthens their analytical thinking and creativity (Bearman et al., 2023; Essien et al., 2024; Hooda et al., 2022). However, recent studies indicate that the retention rate of using GenAI tools is relatively low; users tend to abandon them after initial use (Sequoia Capital, 2023; Tong, 2023). As such, it is crucial to investigate what factors influence students’ continued use of GenAI tools.

Although there has been substantial empirical research on students’ adoption of GenAI tools in higher education (Albayati, 2024; Boubker, 2024; Habibi et al., 2023; Strzelecki, 2023), little research has addressed how students’ capabilities impact their use of these tools. Effectively using GenAI tools requires certain capabilities, including crafting precise prompts, evaluating the accuracy of the information, and adapting it to fit their learning needs (Zhai, 2022). At the core are critical thinking, self-directed learning ability, and AI literacy (Al-Abdullatif & Alsubaie, 2024; Essel et al., 2024; Laak et al., 2024; Dwivedi et al., 2023). AI literacy is indispensable because it is the capability to assess, understand, and operate new AI technologies for obtaining useful information (Essien et al., 2024). Critical thinking is crucial in enabling students to effectively reflect on the relevance and accuracy of the information acquired from GenAI tools (Zhai, 2022). Self-directed learning ability can help students employ the available learning resources to obtain key information and solve problems (Esiyok et al., 2024). Consequently, differences in students’ AI literacy, critical thinking, and self-directed learning ability result in varying levels of the quality of information they can access through GenAI tools (Esiyok et al., 2024; Karatas & Arpaci, 2021). Previous studies have shown that information quality influences students’ satisfaction with their usage of GenAI tools, which in turn affects their intention to continue using GenAI tools (Niu & Mvondo, 2024).

Therefore, there arises a positive feedback cycle for using GenAI tools in higher education. Students with stronger capabilities can not only use GenAI tools to acquire more useful information, but they may also be more inclined to continue using them. Such continuous use of GenAI tools may in turn further improve students’ cognitive skills. While existing studies confirm that GenAI tools positively influence students’ cognitive skills, there is a lack of empirical research on how students’ capabilities affect their use of GenAI tools (Essel et al., 2024). To close the aforementioned research gap, this study aims to answer the following research questions (RQs):

1. What is the relationship between students’ critical thinking, self-directed learning ability, and AI literacy and the quality of information that they acquire from GenAI tools in higher education?
2. What factors influence students’ intention to continue using GenAI tools in higher education, and what is the mediating role of satisfaction and information quality?

This study is structured as follows: Section 2 reviews the relevant literature and proposes the hypotheses; Section 3 explains the research method; Section 4 provides the analysis and results; Section 5 presents the discussion; and Section 6 concludes with key findings and recommendations for future research.

## 2. Literature Review and Hypotheses Development

### 2.1. Theoretical Foundation

This study proposes an analytical framework by combining the Information System Success Model (ISSM) with the Expectation-Confirmation Model (ECM). The ISSM model is a well-accepted theory for explaining usage behavior, developed by DeLone and McLean (1992). Past studies claim that information quality and satisfaction are the crucial determinants of any information system platform’s success (Gao et al., 2015; Veeramootoo et al., 2018). Based on ISSM, several studies explore the usage behaviors of GenAI tools, such as ChatGPT 3.5, in higher education, where information quality and satisfaction are used as the key variables to reveal the underlying mechanisms (Ashfaq et al., 2020; Boubker, 2024; Niu & Mvondo, 2024). Accordingly, in the context of applying GenAI tools in higher education, the current study incorporates the most relevant constructs—information quality and satisfaction—into its conceptual framework.

Additionally, another theoretical foundation of this study is Bhattacherjee’s (2001) ECM for information technology. The ECM has proven to be a successful model in explaining users’ intention to continue using information technology (IT) literature (Gupta et al., 2020; Joo et al., 2017). Previous studies applied the ECM to understand users’ continued use, examining fields from IT to mobile applications (Jenneboer et al., 2022). Ambalov’s (2018) meta-analysis based on 51 ECM studies revealed that the ECM is appropriate for studying users’ satisfaction and continued use. Essentially, satisfaction is the key predictor of continued use (Bhattacherjee, 2001; Bölen, 2020). That is, users who are more satisfied with new technologies are more likely to continue using them.

### 2.2. Hypotheses Development and Conceptual Model

#### 2.2.1. Higher Education Students’ Critical Thinking, Self-Directed Learning Ability, and AI Literacy and Information Quality of GenAI Tools

It is believed that GenAI tools facilitate an iterative dialogue, encouraging students to refine their inquiries and critically reflect on the relevance and accuracy of the information through fact-checking and cross-referencing (Boubker, 2024). Thus, these tools not only allow students to access conveniently information and streamline their learning, but more importantly, they improve students’ abilities and overall academic performance (Boubker, 2024; Essel et al., 2024). The development of GenAI technologies and their applications in higher education has also led to changes in the necessary capabilities of students’ learning accordingly (Almassaad et al., 2024). While interacting with GenAI tools may seem simple—ask a question, obtain an answer—maximizing their potential requires well-crafted prompts to elicit precise, relevant, and personalized responses (Z. Lin, 2024). As a result, certain key skills have become indispensable for students to effectively navigate and leverage these tools. Among these, critical thinking, self-directed learning ability, and AI literacy emerge as must-have skills for students to effectively and responsibly use GenAI tools (Essel et al., 2024; Karatas & Arpaci, 2021).

Critical thinking has long been considered one of the most crucial high-order skills in higher education. Although it is a highly contentious skill and researchers still debate its specific definition, the core elements of the concept remain consistent. Critical thinking mainly refers to an individual’s ability to identify central issues, as well as assumptions, in an argument, make correct inferences from data, evaluate evidence or authority, and make self-corrections, among other skills (Tiruneh et al., 2014). Critical thinking is required to successfully navigate the overwhelming amount of information sources online (Wallace & Jefferson, 2013). In the context of applying GenAI tools, students need to critically reflect on the relevance and accuracy of the information derived from these tools through fact-checking and cross-referencing. Consequently, students’ critical thinking is highly related to the information quality generated by GenAI tools.

Self-directed learning ability is thought to play a central role in professional and educational success (Murane & Levy, 1996; Rees & Bary, 2006). Self-directed learning is defined as a process during which learners identify learning needs, formulate goals, select and apply the appropriate learning sources and strategies, and evaluate their learning outcomes (Knowles, 1975). Students with a high level of self-directed learning ability are self-motivated learners who can employ the available learning resources to solve problems in learning tasks (Brockett & Hiemstra, 1991; Candy, 1991). More importantly, researchers believe that self-directed learners can benefit from online learning environments (Chou, 2012; Morris, 2019). Past studies have revealed that self-directed learning ability is positively associated with students’ usage behaviors of digital technologies, including GenAI tools (Esiyok et al., 2024; Gokcearslan, 2017; Pan, 2020). Therefore, it is reasonable to extrapolate that students with higher self-directed learning ability have a greater chance of refining their inquiries and interactions with GenAI tools, ultimately enabling these tools to exhibit a higher quality of information.

AI literacy is commonly defined as an individual’s ability to understand, use, and apply AI technology ethically (Ng et al., 2021). Before the emergence of AI literacy, the concepts of digital literacy and digital competency were used to evaluate users’ proficiency with computer-related technologies (Leahy & Dolan, 2010). However, with the advent of AI, scholars have argued that previous measures are no longer sufficient (Long et al., 2019). Because this technology enables machines to learn, reason, and perceive in ways that mimic human intelligence, it differs fundamentally from earlier digital technology. Instead, a more nuanced understanding of literacy is required to capture the unique challenges and competencies associated with interacting with AI (Wang et al., 2022). Individuals with higher AI literacy possess the capability to assess, understand, and operate new AI technologies (Long & Magerko, 2020). Accordingly, they may learn to use these technologies more easily (P. Y. Lin et al., 2021). Given that GenAI represents a concrete application within the broader field of AI, AI literacy is particularly suited for assessing the skills needed to effectively use these tools. Previous studies indicate that individuals with higher AI literacy tend to use GenAI tools more effectively, which helps them adopt and learn to improve GenAI tools’ outcomes for specific needs (Lee & Park, 2023; van Dis et al., 2023; Tlili et al., 2023). In this sense, students with higher AI literacy are more likely to obtain higher information quality when using GenAI tools.

Hence, we propose the following:

**H1.**  *Higher education students’ critical thinking is positively associated with the information quality of GenAI tools.*

**H2.**  *Higher education students’ self-directed learning ability is positively associated with the information quality of GenAI tools.*

**H3.**  *Higher education students’ AI literacy is positively associated with the information quality of GenAI tools.*

#### 2.2.2. Information Quality, Satisfaction, and Intention to Continue Using Gen AI Tools

The effective use of GenAI tools requires specific capabilities, which directly influence the quality of information students can obtain from these tools for learning (Laak et al., 2024). Additionally, it is crucial to investigate the factors that influence students’ intention to continue using these tools. This is because, when used effectively and consistently, GenAI tools can enhance the development of students’ capabilities, which allows them to fully realize the potential benefits of GenAI tools. However, recent studies indicate that the retention rate of GenAI tools is relatively low (Sequoia Capital, 2023; Tong, 2023), highlighting the need for a deeper exploration of the determinants of their continued use. Previous research has shown that information quality significantly affects user satisfaction, which in turn influences the intention to continue using GenAI tools (Niu & Mvondo, 2024).

Information quality is defined as the accuracy, relevance, completeness, and timeliness of information produced by digital technologies (Houhamdi & Athamena, 2019; Gao et al., 2015). The ISSM posited that information quality, as the output of an information system, is one of the major components explaining satisfaction, which was validated by numerous studies (Dirgantari et al., 2020; Sayaf, 2023). Previous studies also confirmed the positive relationship between information quality and satisfaction in the usage of GenAI tools, such as ChatGPT (Niu & Mvondo, 2024).

Based on the ISSM, information quality is also a fundamental factor determining whether people will continue to use digital technologies (DeLone & McLean, 2003; Panahifar et al., 2018). When individuals perceive that the information provided by technology is accurate and reliable, they are more likely to trust the technology and keep using it (Namahoot & Laohavichien, 2015). In the educational context, when information provided by a new digital technology is of high quality, students are more likely to use the new technology for their learning purposes (Aparicio et al., 2017). It has been found that the quality of information significantly influences students’ use of ChatGPT, which is the most popular GenAI tool (Boubker, 2024).

According to the ECM, individuals use an information system with the expectation that it will help them accomplish a task, and satisfaction increases when users’ expectations are met (Espejel et al., 2008). It is believed that overall satisfaction is a crucial factor that determines whether users will engage with a certain information system and maintain a high intention to continue using it (Arghashi & Yuksel, 2022). When information is not relevant to the task or is difficult to understand, users may seek alternative methods to obtain the information. By contrast, when an information system provides relevant, reliable, and easily understandable information, it can enhance satisfaction and the intention to continue using it. This has also been confirmed in the usage behavior of GenAI tools (Boubker, 2024; Niu & Mvondo, 2024). To sum up, based on the ISSM and ECM theories, if higher education students obtain higher-quality information, they are possibly more satisfied with these tools, which will finally encourage students to continue using them in the future.

Accordingly, we raise the following hypotheses:

**H4.**  *The information quality of GenAI tools is positively associated with higher education students’ satisfaction with these tools.*

**H5.**  *The information quality of GenAI tools is positively associated with higher education students’ intention to continue using these tools.*

**H6.**  *Higher education students’ satisfaction with these tools is positively associated with their intention to continue using these tools.*

**H7.**  *The information quality of GenAI tools has a positive impact on higher education students’ intention to continue using these tools through the mediation effect of satisfaction with them.*

**Figure 1:**
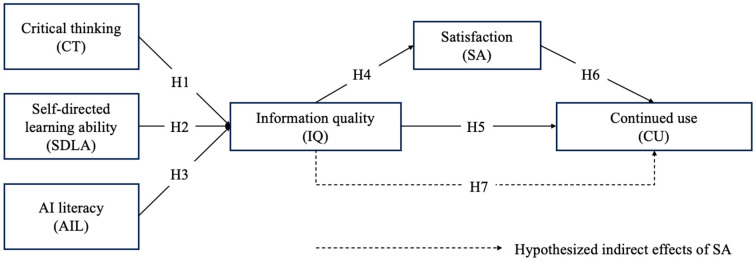
Research model.

Based on the above research aims, the research model of the current study is summarized in Figure 1.

## 3. Methods

### 3.1. Procedure

This study was carried out from 28 December 2023 to 10 January 2024 through an online questionnaire, following a convenience sampling procedure. Questionnaires were distributed among undergraduate and graduate students at 21 universities. The research protocol was approved in advance, and all data were collected anonymously. Before completing the questionnaire, participants could review the informed consent, which detailed the purpose of the study and the safe storage of their data. Participants would only begin completing the questionnaire after agreeing to participate in the study. Out of the 2694 potential respondents, a valid data set of 1784 (66.22% of the responses) participants was obtained.

### 3.2. Participants

**Figure 2:**
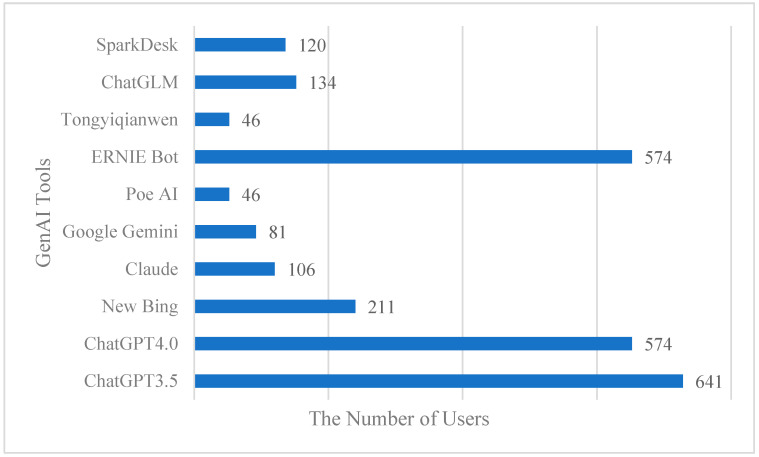
GenAI tools most commonly used by higher education students in China.

The participants in this study were higher education students. Among the valid responses, 1448 (81.12%) used GenAI tools for their studies and research; 54.80% were female, 45.20% were male; 63.80% were undergraduates, 36.20% were graduates; 58.35% came from non-elite universities, 41.64% came from elite universities^1^; and 42.30% majored in science, technology, engineering and mathematics, 57.70% majored in humanities and social science. The percentages of the time when students began frequently using electronic products before entering elementary school, during elementary school, during junior school, during high school, and after entering university or beyond were 8.22%, 40.14%, 29.80%, 12.94%, and 8.19%, respectively. Figure 2 shows the GenAI tools which are most commonly used by higher education students in China.

### 3.3. Instrument

The survey was presented in Chinese and consisted of three sections. The first section contained a standardized instrument adapted from previous studies with critical thinking (ten items) (Stupple et al., 2017), self-directed learning ability (ten items) (Askin-Tekkol & Demirel, 2018; Esiyok et al., 2024), and AI literacy (nine items) (Wang et al., 2022) on a seven-point Likert scale. The second section assessed the use of GenAI tools among higher education students, including information quality (four items), satisfaction (four items), and intention to continue using them (four items), also on a seven-point Likert scale (Ashfaq et al., 2020; Niu & Mvondo, 2024). The scale ranged from 1 (strongly disagree) to 7 (strongly agree). The scale items and results of reliability are shown in Table A1. Cronbach’s alpha estimates for the current sample are all above 0.8, with Composite Reliability (CR) exceeding 0.7 and Average Variance Extracted (AVE) being above 0.5, indicating good evidence for reliability and validity (Nunnally & Bernstein, 1994). The last section captured demographic information, i.e., gender, age, major, university attended, etc.

### 3.4. Data Analysis

Data analysis was performed using SPSS 26.0 and AMOS 26.0. Structural Equation Modeling (SEM), using maximum likelihood to assess the overall research model. Initially, the measurement model was evaluated to confirm its internal consistency, reliability, and convergent validity. Then, the overall fit of the structural model was determined using the Chi-Square index (χ^2^), RMSEA, CFI, and TLI. Once an acceptable fit was achieved, individual parameter estimates were examined to test the hypotheses among the constructs.

## 4. Results

### 4.1. Descriptive Statistics

Table 1 presents the means, standard deviations, t-test results, and correlations for the six variables across the three groups. Notably, the overall mean scores exceed the midpoint, ranging from 5.08 to 5.53. The results of the correlation analysis showed positive relationships between the analyzed variables.

### 4.2. Structural Equation Modeling

Structural equation modeling confirmed the proposed model’s hypothesized relationships. The model fit was strong (χ^2^ = 241.131, df = 141, RMSEA = 0.058, CFI = 0.943, TLI = 0.934). Table 2 lists the standardized path coefficients, with all seven hypotheses supported (coefficients ranging from 0.195 to 0.557).

**Figure 3:**
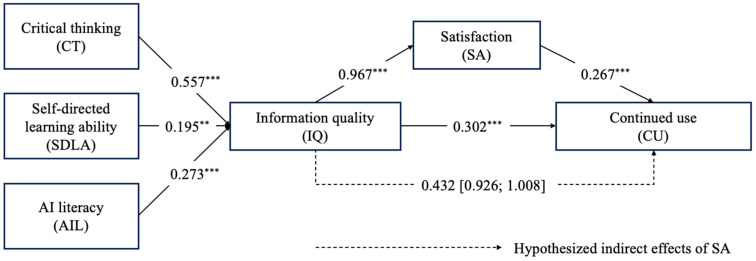
Summarized results of hypothesis testing. Note. ** *p* < 0.01; *** *p* < 0.001.

According to Table 2, critical thinking (β = 0.557, *p* < 0.001), self-directed learning ability (β = 0.195, *p* < 0.01), and AI literacy (β = 0.273, *p* < 0.001) are positively and significantly related to information quality. Therefore, H1, H2, and H3 are supported. Meanwhile, information quality is positively and significantly related to satisfaction (β = 0.967, *p* < 0.001) and intention to continue using GenAI tools (β = 0.302, *p* < 0.001). In addition, there was a positive correlation between satisfaction and intention to continue using GenAI tools (β = 0.267, *p* < 0.001). This supports H4, H5, and H6. Furthermore, information quality has a positive impact on continued use through the mediation of satisfaction (β = 0.432, bootstrap 95% [0.926, 1.008]), which supports H7. The results are summarized in Figure 3.

## 5. Discussion

This study aims to understand how higher education students’ capabilities influence their use of GenAI tools by combining the ISSM and ECM theories. Specifically, this study identifies the factors determining the information quality of GenAI tools obtained by higher education students. Furthermore, this study explores the mechanisms through which information quality and user satisfaction influence higher education students’ continued use of GenAI tools. The seven hypotheses of the proposed research model are supported. The results show that higher education students’ critical thinking, self-directed learning ability, and AI literacy can significantly influence the information quality of GenAI tools. Next, the information quality of GenAI tools, and higher education students’ satisfaction with GenAI tools significantly affect their intention to continue using GenAI tools. Further, information quality also affects higher education students’ satisfaction with GenAI tools. Moreover, satisfaction with GenAI tools mediated the relationship between information quality and higher education students’ intention to continue using GenAI tools.

These findings contribute to our understanding of the integration of GenAI tools in higher education. This is achieved by closing the research gap regarding how students’ capabilities influence their use and continued use of these tools. In line with Esiyok et al. (2024) and Karatas and Arpaci (2021), this study suggests that students’ capabilities—including critical thinking, self-directed learning ability, and AI literacy—are highly significant factors influencing the information quality derived from GenAI tools. Among these capabilities, critical thinking emerged as the most influential factor, suggesting that students who rigorously analyze and evaluate information are more capable of filtering out irrelevant content and extract accurate and useful insights (Boubker, 2024; Essel et al., 2024). At the same time, self-directed learning ability empowers students to independently seek, integrate, and apply new information for studying, thereby refining their queries and optimizing their interaction with GenAI tools (Esiyok et al., 2024). Similarly, AI literacy provides the technical and conceptual foundation necessary to fully exploit the features of GenAI, ensuring that students can effectively interpret and adapt the generated information to their learning needs (van Dis et al., 2023).

Moreover, the current study confirms the relationship between information quality, satisfaction, and intention to continue using GenAI tools. Information quality measures the quality of the content from GenAI tools, which is a crucial factor influencing the satisfaction with these tools and the intention to continue using them (Boubker, 2024; Niu & Mvondo, 2024). When students perceive the information as relevant, reliable, and timely, their overall satisfaction with GenAI tools increases. This heightened satisfaction acts as a driving force that increases the likelihood of continued engagement with these tools. Next, the results of SEM confirm the mediating effects of information quality, satisfaction, and intention to continue using GenAI tools. These relationships collectively suggest the existence of a positive feedback loop as follows: enhanced cognitive and technical competencies lead to improved information quality, which elevates satisfaction and fosters the sustained usage of GenAI tools.

This positive cycle may have long-term implications in terms of higher education students’ capabilities. Previous studies have shown that using GenAI tools can enhance students’ cognitive skills, such as critical thinking (Essien et al., 2024; Jia & Tu, 2024; Wu et al., 2024). This study builds on these findings by demonstrating that students with stronger capabilities are not only more effective in using GenAI tools, but also more inclined to continue using them. As students repeatedly experience high-quality outputs and the consequent satisfaction, they are likely to further develop their capabilities, thereby reinforcing their ability to effectively utilize GenAI tools. Over time, such continuous engagement could contribute to a widening gap between students with strong cognitive and technical skills and those with weaker ones, underscoring the need for targeted educational interventions.

Theoretically, this study extends the ISSM and ECM by exploring the impact of students’ capabilities and the mediating role of user satisfaction between information quality and the intention to continue using GenAI tools. Traditionally used for conventional information systems, these models proved robust in explaining user satisfaction and continued use, even within the context of GenAI. First, this study extends the ISSM theory by confirming users’ abilities (critical thinking, self-directed learning ability, and AI literacy) can influence the information quality of new technology such as GenAI tools. Second, this study combined the ISSM and ECM theories and proposed the mediating role of satisfaction between information quality and continued use, which further illustrated the underlying mechanism. The ISSM theory supports the relationship between information quality and satisfaction, summarized as follows: a higher level of information quality leads to a higher level of satisfaction with using the technology. The ECM theory predicts that satisfaction is a critical determinant of users’ intention to continue using the technology (Bhattacherjee, 2001), which is validated in this study. This reinforces the notion that the quality of the information provided determines long-term engagement.

## 6. Conclusions, Implications, and Limitations

This study adds to our current understanding of how students’ capabilities influence the use of GenAI tools. Although there has been substantial research on the adoption of GenAI tools among higher education students, limited attention has been given to how students’ capabilities shape their effective use of this cutting-edge technology. This study suggests that students with higher levels of critical thinking, self-directed learning ability, and AI literacy are more capable of obtaining relevant information, evaluating its accuracy, and integrating it into their learning processes, leading to the sustained use of GenAI tools. It suggests that the mere availability of GenAI tools is insufficient, as students must possess the necessary cognitive and technical capabilities to fully benefit from these tools. This underscores the significance of equipping students with these key capabilities in a GenAI learning environment, as they are fundamental for maximizing the benefits of such technologies.

This study has significant implications for understanding the role of students’ capabilities in maximizing the potential benefits of GenAI tools in higher education. For higher education institutions and educators, it is crucial to ensure that students are equipped with the capabilities necessary to use these tools. The pronounced role of critical thinking in our findings highlights the necessity for educational institutions to prioritize this ability within their AI literacy curricula. By strengthening students’ critical evaluation skills, educators can better prepare them to navigate and harness the potential of GenAI tools in the long run. However, the interplay between critical thinking, self-directed learning, and AI literacy may produce a synergistic effect that exceeds the sum of their individual impacts. Future research could delve into the potential interaction effects among these capabilities to determine whether there exists a threshold or synergy that amplifies their collective influence on information quality and user satisfaction.

Given the positive relationship between information quality and continued use, educators must prioritize the cultivation of these capabilities for students to access, evaluate, and adapt the content generated by GenAI tools. This is especially pivotal for students with weaker capabilities, who are less likely to continue using GenAI tools, eventually falling into a negative cycle, summarized as follows: lacking capability–ineffective use of GenAI tools–less development of that capability (Essel et al., 2024). More importantly, educators should also guide students in using these tools critically and reasonably. There is concern that over-reliance on GenAI tools may hinder the development of independent problem-solving skills and lead to the uncritical acceptance of GenAI-generated information (Dwivedi et al., 2023). Therefore, it is imperative that future educational interventions not only foster the necessary capabilities for using these tools, but that they also promote responsible and reflective usage practices to ensure that they serve as effective learning aids rather than as crutches.

This study has certain limitations. First, as this research relies on survey data from Chinese higher education students selected via convenience sampling, the findings may not fully represent the broader student population due to the non-random selection process. Second, our reliance on self-reported measures introduces inherent subjectivity to the data. Future studies could benefit from supplementing or triangulating self-report data with more objective metrics, such as usage logs or the content analysis of GenAI-generated outputs, to gain a more comprehensive understanding of how GenAI tools are employed. Additionally, because the study is based on correlational data, it cannot definitively establish causal relationships between the examined variables. To better understand the underlying mechanisms, future research should consider experimental or quasi-experimental designs that can more rigorously assess how changes in GenAI tool usage impact student outcomes. These designs would enable researchers to manipulate key variables and observe their effects over time, thereby providing stronger evidence regarding causal relationships. Incorporating such methodologies could yield a deeper understanding of the mechanisms driving the sustained use of GenAI tools in higher education and help identify more targeted interventions for improving students’ learning experiences.

## Figures and Tables

**Table 1:** Means, standard deviations, and correlations of the analyzed variables.

	Mean (SD)	Critical Thinking	Self-Directed Learning Ability	AI Literacy	Information Quality	Satisfaction
Critical Thinking	5.53 (0.88)					
Self-Directed Learning Ability	5.44 (0.94)	0.80 ***				
AI Literacy	5.39 (0.83)	0.66 ***	0.58 ***			
Information Quality	5.08 (1.04)	0.52 ***	0.55 ***	0.50 ***		
Satisfaction	5.13 (1.03)	0.52 ***	0.54 ***	0.51 ***	0.82 ***	
Continued Use	5.16 (1.05)	0.47 ***	0.50 ***	0.50 ***	0.78 ***	0.91 ***

Note. *** *p* < 0.001.

**Table 2:** Hypothesis testing for the overall model.

Path	Coefficient	S. E	Results
Critical Thinking → Information Quality	0.557 ***	0.079	Supported H1
Self-directed Learning Ability → Information Quality	0.195 **	0.063	Supported H2
AI Literacy → Information Quality	0.273 ***	0.045	Supported H3
Information Quality → Satisfaction	0.967 ***	0.036	Supported H4
Information Quality → Continued Use	0.302 ***	0.044	Supported H5
Satisfaction → Continued Use	0.267 ***	0.040	Supported H6
Information Quality → Satisfaction → Continued Use	0.432 ***	0.141	Supported H7

Note. Coefficient = standardized coefficient; S.E. = standard error; ** *p* < 0.01; *** *p* < 0.001.

## Data Availability

The data set can be made available from the corresponding author(s) upon reasonable request.

## Note

1 In this study, universities supported by the Double-First-Class Plan are considered to be elite universities, while the left are non-elite universities. Double-First-Class Plan: ‘Double’ refers to both university and discipline. ‘First-Class’ refers to the objective of reaching the world-class standard.

## Appendix A

### Questionaire

Factor loadings above 0.50 are recommended (Hair et al., 2019). The results have demonstrated that the factor loadings for all items were above 0.50. A CR value above 0.70 and an AVE greater than 0.50 are considered adequate (Nunnally & Bernstein, 1994). As Table A1 illustrates, the values of CR and AVE for all constructs reached the threshold.

**Table A1:** Questionnaire items in English and in Chinese and the results of reliability.

Construct	Item	Standardized Factor Loadings
Critical Thinking (α = 0.91; AVE = 0.52; CR = 0.91)		
CT1	I believe that when addressing issues, it is essential to identify their root causes. 我认为处理问题时，首先要弄清楚问题的根本原因	0.71
CT2	I am able to grasp the essence and key points of matters. 我能够抓住事物的本质和重点	0.71
CT3	Before making an important decision, I strive to gather relevant information. 在作一个重要决策前，我会尽力搜集有关资料	0.69
CT4	I can propose innovative solutions to problems. 我能够提出有创见性的解决问题的方法	0.73
CT5	When encountering a new theory, I examine its validity from multiple perspectives. 在接受一个新理论时，我会从多方面考察其合理性	0.76
CT6	While learning new knowledge, I consider employing different methods. 在学习新知识时，我会考虑使用不同的方法	0.72
CT7	Organizing my thoughts is an easy task for me. 对我来说，组织好自己的思路是件很容易的事	0.73
CT8	Even when dealing with complex issues, I always maintain clear thinking. 即便是处理复杂问题我也总是思路很清晰	0.71
CT9	When evaluating other people’s opinions, I highly value whether there is reliable evidence supporting them. 当别人发表观点时，我非常看重有没有可靠的证据支撑	0.73
CT10	I draw conclusions only when there is conclusive evidence. 在有确凿证据的情况下，我才会对事情下结论	0.72
Self-directed Learning Ability (α = 0.92; AVE = 0.57; CR = 0.92)		
SDLA1	I can set appropriate learning goals based on my needs and abilities. 我能根据自己的学习需求和能力确立合适的学习目标	0.77
SDLA2	When studying, I develop a general learning plan to enhance my learning effectiveness. 学习一门课程或者技能时，我都会制定大致的学习计划以提高自己学习的效果	0.78
SDLA3	I can effectively implement my learning plans to achieve the expected objectives. 我能够有效执行自己的学习计划，实现预期学习目标	0.71
SDLA4	I can find valuable resources according to my learning needs. 我能依据自己的学习需要找到有价值的学习资源	0.79
SDLA5	I can arrange my study time reasonably. 我能够合理安排学习时间	0.80
SDLA6	I can choose effective learning methods. 我能够选择有效的学习方法	0.75
SDLA7	I can stay focused when studying. 我能够在学习时保持专注	0.74
SDLA8	I can reflect on problems in learning process and take timely corrective actions. 我能够反思自己在学习过程中存在的问题，及时补救	0.78
SDLA9	I adjust my learning plans based on the situation. 我会根据自己的实际情况调整学习计划	0.75
SDLA10	I can reasonably evaluate my learning outcomes. 我能够合理地评价自己的学习效果	0.73
AI Literacy (α = 0.89; AVE = 0.60; CR = 0.90)		
AIL1	I understand the concepts related to AI. 我了解人工智能的基本概念	0.70
AIL2	I am familiar with the application of AI. 我了解应用人工智能技术的产品	0.63
AIL3	I am interested in exploring AI and its applications. 我对人工智能技术及其应用有强烈的探究兴趣	0.74
AIL4	I am able to select appropriate AI applications based on my needs. 我能够根据自己的需求选择合适的人工智能产品	0.80
AIL5	I can adapt to technical advancements in AI. 我能适应人工智能技术的变化与发展	0.83
AIL6	I am confident in my ability to use AI to solve problems. 我能够使用人工智能产品解决问题	0.83
AIL7	I understand the risks of AI, such as the potential for erroneous information and data bias. 我知道人工智能可能带来错误信息、数据偏见等风险	0.76
AIL8	I believe that the development of AI should be regulated by ethical guidelines. 我认为人工智能的发展需要伦理规范	0.76
AIL9	I understand that improper use of AI can lead to serious security issues. 我知道如果人工智能使用不当，或被非法滥用，将会造成严重的数据、隐私泄露等安全问题	0.71
Information Quality (α = 0.87; AVE = 0.57; CR = 0.86)		
IQ1	The information provided by GenAI tools is applicable. 生成式AI工具提供给我的信息与我的需求相关	0.85
IQ2	The information provided by GenAI tools is credible. 我认为生成式AI工具提供的信息是可靠的	0.70
IQ3	The information provided by GenAI tools is well presented. 我认为生成式AI工具做到了以恰当的方式呈现信息	0.79
IQ4	GenAI tools provide prompt information. 生成式AI工具能够迅速为我提供信息	0.71
Satisfaction (α = 0.90; AVE = 0.57; CR = 0.86)		
SA1	Overall, I am satisfied with GenAI tools. 我对自己使用生成式AI工具的情况感到很满意	0.81
SA2	GenAI tools have improved my learning efficiency. 生成式AI工具提高了我的学习效率	0.78
SA3	GenAI tools meet my expectations. 生成式AI工具的使用效果达到了我的预期	0.71
SA4	GenAI tools help me address my problems. 我通过生成式AI工具获得的信息能够解决我的问题	0.68
Continued Use (α = 0.90; AVE = 0.57; CR = 0.84)		
CU1	I will continue to use GenAI tools. 我愿意继续使用生成式AI工具	0.69
CU2	I plan to increase my use of GenAI tools in the future. 未来我打算增加使用生成式AI工具的频率	0.72
CU3	I am willing to learn how to use GenAI tools more effectively. 我愿意学习如何更有效地使用生成式AI工具	0.81
CU4	I will recommend other people to use GenAI tools. 我会推荐我身边的人使用生成式AI工具	0.77
